# Identification of a Novel Osteogenetic Oligodeoxynucleotide (osteoDN) That Promotes Osteoblast Differentiation in a TLR9-Independent Manner

**DOI:** 10.3390/nano12101680

**Published:** 2022-05-14

**Authors:** Yuma Nihashi, Mana Miyoshi, Koji Umezawa, Takeshi Shimosato, Tomohide Takaya

**Affiliations:** 1 Department of Science and Technology, Graduate School of Medicine, Science and Technology, Shinshu University, 8304 Minami-minowa, Kami-ina, Nagano 399-4598, Japan; 19hs504f@shinshu-u.ac.jp (Y.N.); shimot@shinshu-u.ac.jp (T.S.); 2 Department of Agriculture, Graduate School of Science and Technology, Shinshu University, 8304 Minami-minowa, Kami-ina, Nagano 399-4598, Japan; 21as121c@shinshu-u.ac.jp; 3 Department of Agricultural and Life Sciences, Faculty of Agriculture, Shinshu University, 8304 Minami-minowa, Kami-ina, Nagano 399-4598, Japan; koume@shinshu-u.ac.jp; 4 Department of Biomolecular Innovation, Institute for Biomedical Sciences, Shinshu University, 8304 Minami-minowa, Kami-ina, Nagano 399-4598, Japan

**Keywords:** osteogenetic oligodeoxynucleotide (osteoDN), osteoblast, bone differentiation, calcification, mineralization, Toll-like receptor 9 (TLR9)

## Abstract

Dysfunction of bone-forming cells, osteoblasts, is one of the causes of osteoporosis. Accumulating evidence has indicated that oligodeoxynucleotides (ODNs) designed from genome sequences have the potential to regulate osteogenic cell fate. Such osteogenetic ODNs (osteoDNs) targeting and activating osteoblasts can be the candidates of nucleic acid drugs for osteoporosis. In this study, the ODN library derived from the *Lacticaseibacillus rhamnosus* GG genome was screened to determine its osteogenetic effect on murine osteoblast cell line MC3T3-E1. An 18-base ODN, iSN40, was identified to enhance alkaline phosphatase activity of osteoblasts within 48 h. iSN40 also induced the expression of osteogenic genes such as Msx2, osterix, collagen type 1α, osteopontin, and osteocalcin. Eventually, iSN40 facilitated calcium deposition on osteoblasts at the late stage of differentiation. Intriguingly, the CpG motif within iSN40 was not required for its osteogenetic activity, indicating that iSN40 functions in a TLR9-independent manner. These data demonstrate that iSN40 serves as a novel osteogenetic ODN (osteoDN) that promotes osteoblast differentiation. iSN40 provides a potential seed of the nucleic acid drug that activating osteoblasts for osteoporosis therapy.

## 1. Introduction

The bone is a metabolically active organ that remodels continuously throughout life. However, bone remodeling function is impaired with aging, resulting in reduced bone mass and micro-architectural deterioration [1]. It finally causes osteoporosis and increases fracture risk, which is related to around 200 million people and annually 8.9 million fractures in the world [2]. One of the pathological events expressing osteoporosis is the dysfunction of bone-forming cells, osteoblasts. Differentiation from osteoblasts into osteocytes is regulated by canonical Wnt/β-catenin signaling pathway [3]. It stimulates two bone-specific transcription factors, Runx2 and osterix [4], which coordinately regulate the expression of osteogenic genes such as collagen type 1α, osteopontin, osteocalcin, and receptor activator of NF-κB ligand (RANKL) [5]. However, osteoblasts of elderly people present dysregulated expression of these bone-related genes [6,7,8], indicating the decreased bone-forming capacity of aged osteoblasts. Therefore, re-activation of osteoblasts is a key therapeutic strategy for osteoporosis.

Several agents have been developed to regulate osteoblast function [9]. Teriparatide, a parathyroid hormone (PTH) fragment, directly targets PTH receptor on osteoblasts to induce osteoprotegerin and activate bone anabolism [10]. Denosumab, a decoy receptor for RANKL, increases bone mineralization by preventing bone resorption [11]. Currently, it is the most preferred treatment for post-menopausal osteoporosis [12]. Sclerostin is an inhibitor of Wnt signaling [13]. Thus, romosozumab, a sclerostin antibody, activates the Wnt pathway to stimulate bone formation [14]. Their beneficial effects in osteoporosis therapy have been robustly proven. However, with the current technology, these protein/peptide-based drugs are expensive and unstable to be produced in large quantities for the treatment of a large number of osteoporosis patients all over the world. To overcome this problem, other types of molecules targeting osteoblasts have been studied.

As nucleic acids offer several advantages, such as chemical synthesis, low-cost manufacturing, and stability during storage, they are potential nanomolecules for next-generation drugs. A wide variety of nucleotides have been clinically applied; antisense nucleotides that regulate gene expression [15], aptamers that target proteins [16], and ligands of Toll-like receptors (TLRs) that modulate the innate immune system [17] can specifically access their diverse targets. Furthermore, some oligodeoxynucleotides (ODNs) have been reported to regulate osteogenic cell fate. A 27-base cytosine (C)-rich ODN designed from the human mitochondrial genome, named MT01 ([ACC CCC TCT]_3_), was initially identified as an immunosuppressive ODN that inhibits the proliferation of human peripheral blood mononuclear cells [18]. Intriguingly, MT01 promoted the proliferation and differentiation of the human osteoblast-like cell line MG63 [19], rat bone marrow mesenchymal stem cells [20], and murine osteoblast cell line MC3T3-E1 [21] by upregulating Runx2 phosphorylation via activation of the ERK/MAPK pathway [22]. Thus, MT01 treatment reduced alveolar bone loss in rat periodontitis in vivo [20]. These studies suggest that genomic ODNs could be used as drug seeds for bone regeneration.

For muscle, an ODN library designed from the genome sequence of the lactic acid bacterium *Lacticaseibacillus rhamnosus* GG [23] has been screened to determine its effect on myogenic cell fate. The 18-base guanine (G)-rich ODN, iSN04, was found to promote differentiation and suppress inflammation of myogenic precursor cells, myoblasts [24,25,26,27,28]. iSN04, termed myogenetic ODN (myoDN), formed a globular structure of 1-nm radius and served as an anti-nucleolin aptamer in a TLR-independent manner [24]. The discovery of myoDN demonstrates that bacterial genome sequences are promising platforms for providing novel ODNs that can control cell differentiation. To authenticate this concept, this study explored the ODN library used for myoDN study to identify an osteogenetic ODN (osteoDN) that facilitates the differentiation and calcification of osteoblasts.

## 2. Materials and Methods

### 2.1. Chemicals

ODN sequences used in this study are listed in Table S1. Phosphorothioated (PS) ODNs and 6-carboxyfluorecein (6-FAM)-conjugated PS-ODNs were synthesized and purified using HPLC (GeneDesign, Osaka, Japan). PS-ODNs and ascorbic acid (AA) (Fujifilm Wako Chemicals, Osaka, Japan) were dissolved in endotoxin-free water. An equal volume of solvent was used as negative control.

### 2.2. Cell Culture

MC3T3-E1 cell line (RCB1126) was provided by the RIKEN BRC (Tsukuba, Japan) through the Project for Realization of Regenerative Medicine and the National Bio-Resource Project of the MEXT, Japan. The cells were cultured at 37 °C with 5% CO_2_ throughout the experiments. Undifferentiated MC3T3-E1 cells were maintained in a growth medium (GM) consisting of α-MEM (Nacalai, Osaka, Japan), 10% fetal bovine serum (FBS) (HyClone; GE Healthcare, Chicago, UT, USA), and a mixed solution of 100 units/mL penicillin and 100 μg/mL streptomycin (P/S) (Nacalai). To induce osteogenic differentiation, confluent MC3T3-E1 cells were cultured in a differentiation medium (DM) consisting of α-MEM, 10% FBS, 10 nM dexamethasone (Fujifilm Wako Chemicals), 10 mM β-glycerophosphate (Nacalai), and P/S. To facilitate differentiation, 15 or 50 μg/mL AA was added to GM or DM as required.

### 2.3. Alkaline Phosphatase (ALP) Staining

For screening, MC3T3-E1 cells were seeded on 96-well plates (1.0 × 10^4^ cells/100 μL GM/well) for screening or on 12-well plates (1.0 × 10^4^ cells/well) for high-resolution imaging. The next day, the medium was replaced with GM or DM containing 10 μM PS-ODNs. After 48 h, ALP enzymatic activity of the cells was visualized using ALP Stain Kit (Fujifilm Wako Chemicals) according to the manufacturer’s instruction. Phase-contrast images were obtained using EVOS FL Auto microscope (AMAFD1000; Thermo Fisher Scientific, Waltham, MA, USA). ALP-positive area was quantified using ImageJ software version 1.52a (Wayne Rasband; National Institute of Health, Bethesda, MD, USA).

### 2.4. Alizarin Staining

MC3T3-E1 cells were seeded on 24-well plates (3.0–5.0 × 10^4^ cells/well) and cultured in GM until they become confluent. The medium was then replaced with DM containing PS-ODNs every 3–4 days. The cells were fixed with 2% paraformaldehyde and stained with 1% *w*/*v* alizarin red S (Fujifilm Wako Chemicals). Bright-field images were captured using EVOS FL Auto microscope. Alizarin-positive area was quantified using ImageJ.

### 2.5. Quantitative Real-Time RT-PCR (qPCR)

MC3T3-E1 cells were seeded on 60-mm dishes (3.0 × 10^5^ cells/dish). The next day, the medium was replaced with GM or DM containing 10 μM iSN40. Total RNA was isolated using NucleoSpin RNA Plus (Macherey-Nagel, Düren, Germany) and reverse transcribed using ReverTra Ace qPCR RT Master Mix (TOYOBO, Osaka, Japan). qPCR was performed using GoTaq qPCR Master Mix (Promega, Madison, WI, USA) with the StepOne Real-Time PCR System (Thermo Fisher Scientific). The amount of each transcript was normalized to that of the 3-monooxygenase/tryptophan 5-monooxygenase activation protein zeta gene (*Ywhaz*) and presented as fold-changes. The primer sequences are listed in Table S2.

### 2.6. Trivial Trajectory Parallelization of Multicanonical Molecular Dynamics (TTP-McMD)

Starting with the simulation of iSN40 and MT01 structures built from their DNA sequences using NAB in AmberTools [29], an enhanced ensemble method, TTP-McMD [30], was used to sample the equilibrated conformations at 310 K. In the TTP-McMD, energy range of the multicanonical ensemble covered a temperature range from 280 K to 380 K. Sixty trajectories were used, and the production run was conducted for 40 ns in each trajectory (total 2.4 μs). Throughout the simulation, amber ff12SB force field [31] was used, whereas the solvation effect was represented by the generalized-born model [32].

### 2.7. Statistical Analysis

The results are presented as the mean ± standard error. Statistical comparisons between two groups were performed using unpaired two-tailed Student’s *t*-test and among multiple groups using one-way analysis of variance followed by Scheffe’s *F*-test. The statistical significance at *p* values are indicated in all the figures.

## 3. Results

### 3.1. iSN40 Promotes Osteoblast Differentiation

**Figure 1:**
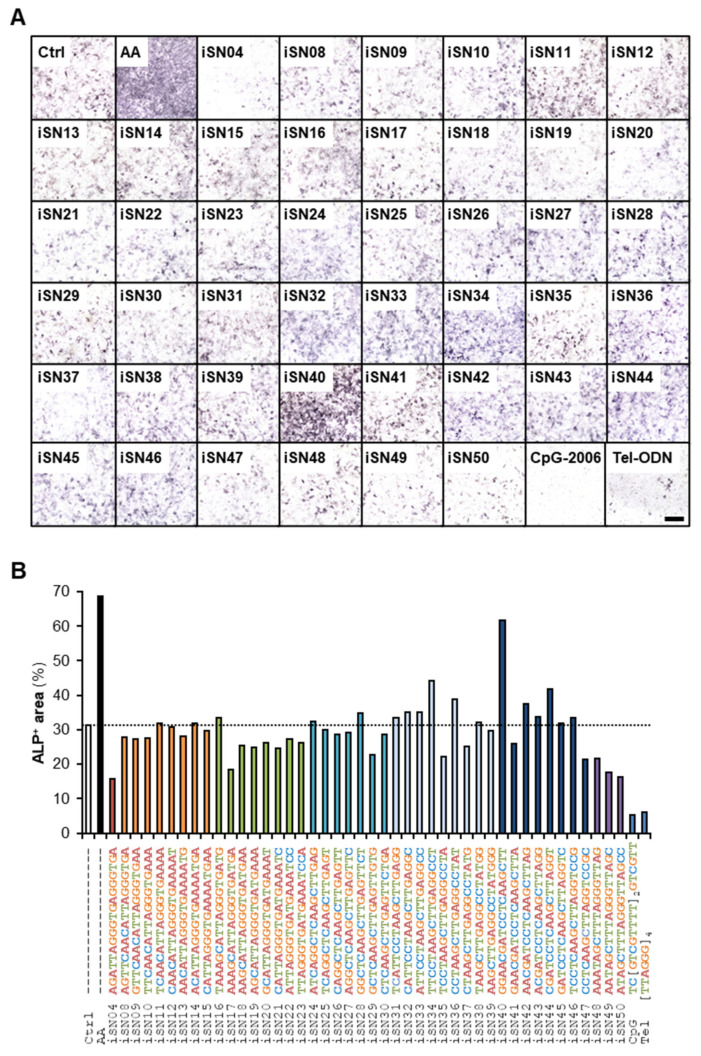
Identification of iSN40 as an osteoDN. (**A**) ALP staining images of MC3T3-E1 cells treated with 10 μM PS-ODNs in GM for 48 h. Scale bar, 200 μm. (**B**) Quantification of ALP-positive area.

Forty-four 18-base PS-ODNs designed from the *Lacticaseibacillus rhamnosus* GG genome (iSN04 and iSN08-iSN50) were administered to MC3T3-E1 cells. In addition, two immunomodulatory PS-ODNs were concomitantly tested: CpG-2006 is a TLR9 ligand that initiates inflammatory cascades [33], and Tel-ODN is a human telomeric ODN that suppresses immunological reactions depending on TLR3/7/9 [34]. The osteogenic effects of these PS-ODNs were investigated by measuring ALP enzymatic activity in the cells, a standard marker of osteoblast differentiation (Figure 1A). iSN40 (GGA ACG ATC CTC AAG CTT) markedly increased ALP-positive area to the same extent as AA, positive control for promoting osteogenic differentiation (Figure 1B). However, other PS-ODNs did not enhance ALP activity, indicating sequence-dependent osteogenetic activity of iSN40. iSN40-induced ALP activity was reproducibly confirmed by high-resolution images of the experiment performed independently of the screening (Figure S1). These results demonstrate that iSN40 serves as an osteoDN that promotes osteoblast differentiation.

### 3.2. iSN40 Modulates Osteogenic Gene Expression

**Figure 2:**
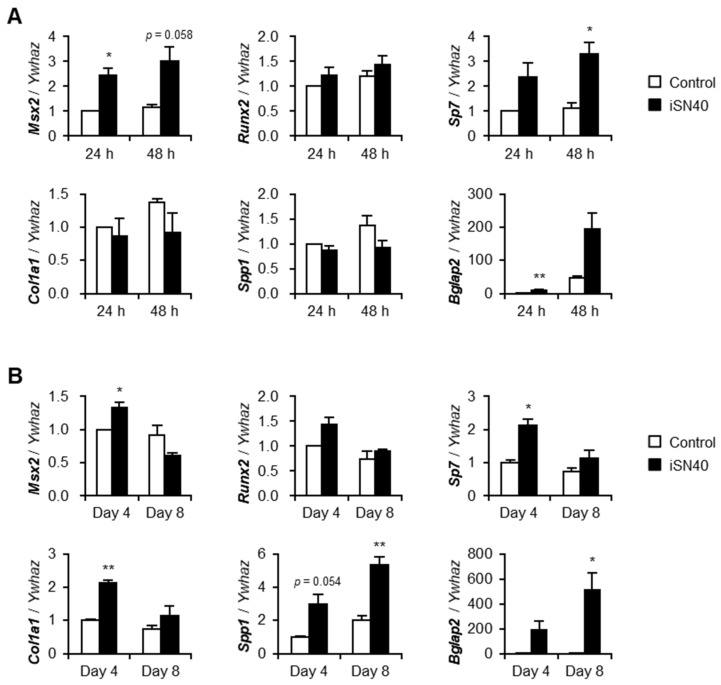
iSN40 induces osteogenic gene expression in MC3T3-E1 cells. (**A**) qPCR results of MC3T3-E1 cells treated with 10 μM iSN40 in GM with 15 μg/mL AA for 24 or 48 h. (**B**) qPCR results of MC3T3-E1 cells treated with 10 μM iSN40 in DM with 15 μg/mL AA for 4 or 8 days. * *p* < 0.05, ** *p* < 0.01 vs. control at each time point (Student’s *t* test). *n* = 3.

The effect of iSN40 on osteogenic gene expression in MC3T3-E1 cells was investigated by qPCR. To analyze the early stage of differentiation, MC3T3-E1 cells were treated with iSN40 in GM containing AA for 24–48 h (Figure 2A). iSN40 increased the mRNA levels of Msx2, a homeobox transcription factor that promotes osteogenesis through bone morphogenic protein 2-induced signaling. In contrast, iSN40 did not alter the levels of Runx2, a master regulator that determines osteoblast lineage and inhibits bone maturation [35]. Interestingly, iSN40 significantly upregulated the expression of osterix (*Sp7*), a downstream target of Runx2. iSN40 did not improve the mRNA levels of immature myoblast markers, collagen type 1α (*Col1a1*) and osteopontin (*Spp1*); however, iSN40 markedly induced osteocalcin (*Bglap2*), a hormone released from the bone [36]. To investigate the late stage of differentiation, MC3T3-E1 cells were treated with iSN40 in DM containing AA for 4–8 days (Figure 2B). iSN40 significantly elevated the levels of Msx2 on day 4 but not that of Runx2. Notably, by day 8, iSN40 upregulated the expression of osterix, collagen type 1α, osteopontin, and osteocalcin, which act downstream of Runx2 [37]. Compared with the results at the early stage, the effect of iSN40 on these gene expression was more significant at the late stage, suggesting a long-term activity of iSN40. These data indicate that iSN40 facilitates osteoblast differentiation by modulating osteogenic gene expression through the transcription factor Msx2 rather than Runx2.

### 3.3. iSN40 Promotes Osteoblast Calcification

**Figure 3:**
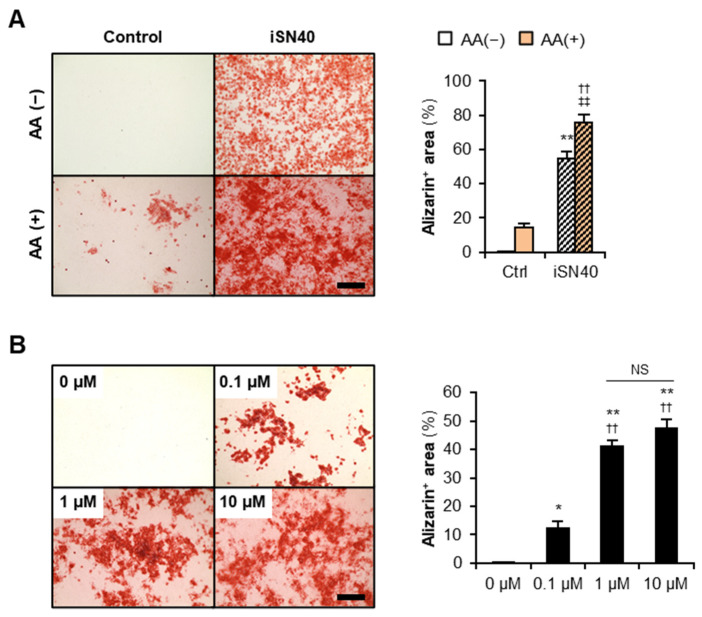
iSN40 induces calcification of MC3T3-E1 cells. (**A**) Representative images and quantification of alizarin staining of MC3T3-E1 cells treated with 10 μM iSN40 in DM with or without 15 μg/mL AA for 12 days. Scale bar, 200 μm. ** *p* < 0.01 vs. control-AA(−), ^††^ *p* < 0.01 vs. control-AA(+), ^‡‡^ *p* < 0.01 vs. iSN40-AA(−) (Scheffe’s *F*-test). *n* = 4. (**B**) Representative images and quantification of alizarin staining of MC3T3-E1 cells treated with 0.1, 1, or 10 μM iSN40 in DM with 15 μg/mL AA for 9 days. Scale bar, 200 μm. * *p* < 0.05, ** *p* < 0.01 vs. 0 μM; ^††^ *p* < 0.01 vs. 0.1 μM; NS, not significant (Scheffe’s *F*-test). *n* = 4.

To investigate the effect of iSN40 on osteoblast calcification, MC3T3-E1 cells were treated with iSN40 in DM with or without AA for 12 days and then subjected to alizarin staining to visualize calcium deposition on the cells (Figure 3A). Alizarin-positive area was significantly increased by 10 μM iSN40 rather than by 15 μg/mL AA. Moreover, compared with individual treatments, co-treatment with iSN40 and AA further facilitated calcification, indicating that iSN40 and AA synergistically promote osteoblast mineralization. Although iSN40 was administered at a concentration of 10 μM in the above experiments, the dose response analysis revealed that 1 μM iSN40 was sufficient to induce osteoblast calcification in the presence of 15 μg/mL AA (Figure 3B). We used phosphorothioated iSN40 (PS-iSN40) in this study to avoid degradation by nucleases, whereas native iSN40 did not promote calcification even in the presence of 50 μg/mL AA (Figure S2). This demonstrates that phosphorothioation is necessary for iSN40 to function as an osteoDN.

### 3.4. Osteogenetic Action of iSN40 Is TLR9-Independent

**Figure 4:**
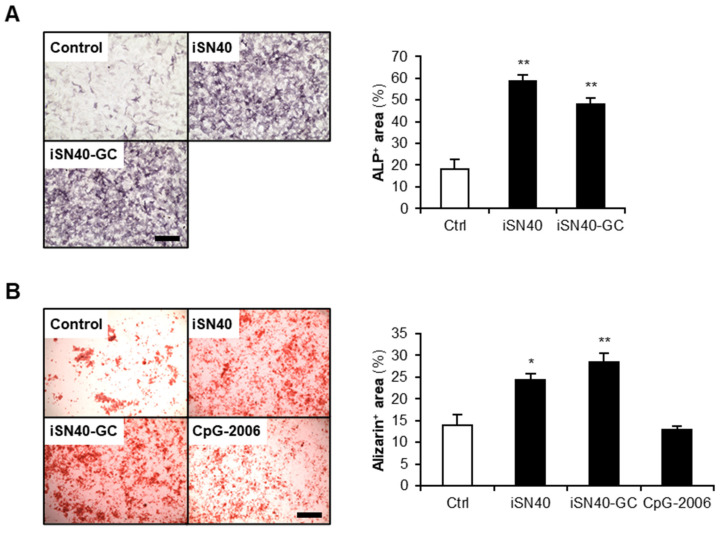
Action of iSN40 is TLR9-independent. (**A**) Representative images and quantification of ALP staining of MC3T3-E1 cells treated with 10 μM of iSN40 or iSN40-GC in DM with 50 μg/mL AA for 48 h. Scale bar, 100 μm. ** *p* < 0.01 vs. control (Scheffe’s *F*-test). *n* = 4. (**B**) Representative images and quantification of alizarin staining of MC3T3-E1 cells treated with 1 μM PS-ODNs in DM with 50 μg/mL AA for 9 days. Scale bar, 200 μm. * *p* < 0.05, ** *p* < 0.01 vs. control (Scheffe’s *F*-test). *n* = 4.

iSN40 (GGA ACG ATC CTC AAG CTT) contained a CpG motif. It has been studied that ODNs possessing unmethylated CpG motifs (CpG-ODNs) serve as TLR9 ligands and initiate innate immune system-induced inflammatory responses [38]. To examine the impact of the CpG motif within iSN40 on its osteogenetic activity, iSN40-GC (GGA AGC ATC CTC AAG CTT) was constructed, in which the CpG motif was substituted with GC. iSN40-GC enhanced ALP activity and calcification of MC3T3-E1 cells to the same extent as iSN40 (Figure 4). Conversely, a well-known TLR9 ligand, CpG-2006 [33], did not facilitate osteoblast mineralization (Figure 4B), which was further confirmed through ALP staining results (Figure 1 and Figure S1). In addition, RT-PCR revealed that MC3T3-E1 cells do not express TLR9 (Figure S3), corresponding to the previous study [39]. These results demonstrate that osteogenetic action of iSN40 is independent of its CpG motif and TLR9.

## 4. Discussion

The present study identified that iSN40, which is an 18-base ODN derived from the genome sequence of lactic acid bacteria, serves as an osteoDN that promotes differentiation and mineralization of osteoblasts. It could be the candidate of nucleic acid drug activating osteoblasts for osteoporosis. Although a direct target and action mechanism of iSN40 are unknown, the study results revealed that the osteogenetic function of iSN40 is TLR9-independent. The CpG motif within iSN40 was not required for its activity; A validated TLR9 ligand, CpG-2006 [33], enhanced neither ALP activity nor calcification of MC3T3-E1 cells; moreover, TLR9 was not expressed in MC3T3-E1 cells. TLR9-independent effect of iSN40 is crucial for its clinical application. The innate immune system, including IL-6 secretion, is closely related to bone homeostasis [40]. For example, CpG-ODNs generally upregulate interleukin (IL)-6 production via TLR9 [17]. IL-6 is a pro-osteoclastogenic cytokine [3] that is highly produced in aged osteoblasts [7]. Therefore, TLR9-independent osteogenetic activity of iSN40 is a favorable characteristic for osteoporosis therapy. However, it is still ambiguous whether iSN40 never serves as a CpG-ODN even in TLR9-expressing cells. Although human osteoblasts and bone-derived mesenchymal stem cells do not express TLR9 as MC3T3-E1 cells do not [41], other types of cells existing in bone tissue such as osteoclasts express TLR9 as a mediator of bone metabolism [42]. Thus, immunological and pro-inflammatory effects of iSN40 on TLR9-expressing cells in bone tissue need to be elucidated for future application in clinical settings.

iSN40 upregulated the expression of osteogenic genes such as osterix, collagen type Iα, osteopontin, and osteocalcin, which are induced by Runx2 [37]. However, iSN40 did not alter Runx2 expression. The transcriptional activity of Runx2 is regulated via post-transcriptional modifications including phosphorylation, acetylation, sumoylation, and ubiquitination, which are mediated by numerous factors [43]. iSN40 may target such modulators to enhance Runx2 activity. For instance, another osteoDN, MT01, has been reported to enhance Runx2 phosphorylation via activation of ERK and p38 MAPK [22], which potentiates the Runx2/osterix transcriptional machinery [44]. The effect of iSN40 on Runx2 protein needs to be examined in further studies.

**Figure 5:**
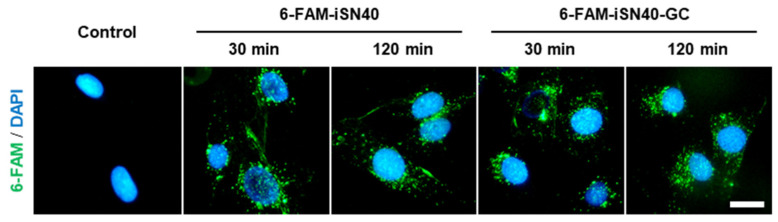
Intracellular incorporation of iSN40. Representative fluorescent images of MC3T3-E1 cells treated with 5 μg/mL 6-FAM-iSN40 and 6-FAM-iSN40-GC in GM for 30- or 120-min. Scale bar, 25 μm.

iSN41-iSN47 are PS-ODNs analogous to iSN40; however, they did not enhance ALP activity of MC3T3-E1 cells. When iSN40 operates as an antisense nucleotide, iSN41-iSN47 would present partial osteogenetic activities. To discuss the mechanism of action of iSN40, a previous study reporting iSN04 is helpful. iSN04 is designed from the lactic acid bacteria, genome as same as iSN40, and serves as a myoDN that promotes myoblast differentiation in a TLR-independent manner [24]. iSN04 is taken up into cytoplasm without any carriers, forms a G-stacking structure, and physically interacts with nucleolin to interfere its function, indicating that iSN04 is an anti-nucleolin aptamer [24]. Thus, it is sufficiently possible that iSN40 works as an aptamer for target proteins. Administration of 6-FAM-conjugated iSN40 and iSN40-GC to MC3T3-E1 cells showed that they were autonomously incorporated into the cytoplasm within 30 min (Figure 5), suggesting that iSN40 acts intracellularly, not on the plasma membrane.

**Figure 6:**
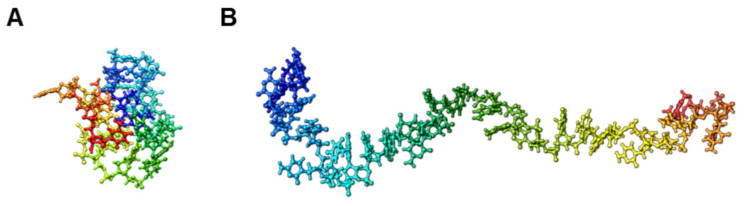
The most thermodynamically stable conformations of the ODNs in water at 310K simulated by the TTP-McMD. (**A**) iSN40. (**B**) MT01.

To further discuss the potential of iSN40 as an aptamer, conformation of iSN40 in water at 310 K was simulated using the TTP-McMD (Figure 6A). iSN40 exhibited a compact globular structure (average radius: 1.01 nm) similar to that of iSN04 [24]. Interestingly, the predicted structure of another osteoDN, MT01, was substantially different from that of iSN40 (Figure 6B), suggesting that the action mechanisms of iSN40 and MT01 are likely to be dissimilar. Indeed, iSN40 did not alter the mRNA levels of Runx2, but MT01 significantly induced Runx2 transcription [19,20]. To determine whether iSN40 is an aptamer, the iSN40-binding protein in osteoblasts needs to be identified. This would further promote the development and application of iSN40 as an osteoDN for bone regeneration.

## 5. Conclusions

The present study successfully identified a novel osteoDN, iSN40, which is an 18-base ODN designed from the lactic acid bacteria genome. iSN40 promoted the differentiation and calcification of osteoblasts by modulating osteogenic gene expression in a TLR9-independent manner. It demonstrated that the ODN library derived from bacteria genome can be a platform to discover the ODN targeting and regulating osteoblasts. Activation of osteoblasts by ODNs would provide an alternative strategy for osteoporosis therapy by promoting bone formation and mineralization.

## 6. Patents

Shinshu University has been assigned the invention of osteoDN by T.T., Y.N., K.U., and T.S., and Japan Patent Application 2021-122713 has been filed on 27 July 2021.

## Data Availability

The raw data supporting the conclusions of this article will be made available by the authors, without undue reservation.

## Supplementary Materials

The Supplementary Materials are available online at: https://www.mdpi.com/article/10.3390/nano12101680/s1. Figure S1: iSN40 enhances ALP activity in MC3T3-E1 cells, Figure S2: Phosphorothioation is required for iSN40 to exert osteogenetic activity, Figure S3: Expression of TLR genes in MC3T3-E1 cells, Table S1: ODN sequences, Table S2: Primer sequences for RT-PCR. References [45,46,47,48,49,50] are cited in the Supplementary Materials.
