# Supplementary material for: Identification of a Novel Osteogenetic Oligodeoxynucleotide (osteoDN) That Promotes Osteoblast Differentiation in a TLR9-Independent Manner
Source: Nanomaterials (Basel). 2022 May 14;12(10):1680. doi: 10.3390/nano12101680 (PMC9145662; doi:10.3390/nano12101680)
Supplement: Supplementary file 1 [file nanomaterials-12-01680-s001.zip › nanomaterials-1686042-supplementary.pdf]

nanomaterials

Supplementary Materials

Identification of a Novel Osteogenetic Oligodeoxynucleotide (osteoDN) that Promotes Osteoblast Differentiation in a TLR9-Independent Manner

Yuma Nihashi [1,†], Mana Miyoshi [2], Koji Umezawa [3,4], Takeshi Shimosato [1,2,3,4] and Tomohide Takaya [1,2,3,4,*]

1 Department of Science and Technology, Graduate School of Medicine, Science and Technology, Shinshu University, 8304 Minami-minowa, Kami-ina, Nagano 399-4598, Japan; 19hs504f@shinshu-u.ac.jp (Y.N.); shimot@shinshu-u.ac.jp (T.S.)
2 Department of Agriculture, Graduate School of Science and Technology, Shinshu University, 8304 Minami-minowa, Kami-ina, Nagano 399-4598, Japan; 21as121c@shinshu-u.ac.jp
3 Department of Agricultural and Life Sciences, Faculty of Agriculture, Shinshu University, 8304 Minami-minowa, Kami-ina, Nagano 399-4598, Japan; koume@shinshu-u.ac.jp
4 Department of Biomolecular Innovation, Institute for Biomedical Sciences, Shinshu University, 8304 Minami-minowa, Kami-ina, Nagano 399-4598, Japan
* Correspondence: ttakaya@shinshu-u.ac.jp
† Current address: Cellular and Molecular Biotechnology Research Institute, National Institute of Advanced Industrial Science and Technology, Central 5-41, 1-1-1 Higashi, Tsukuba, Ibaraki 305-8565, Japan

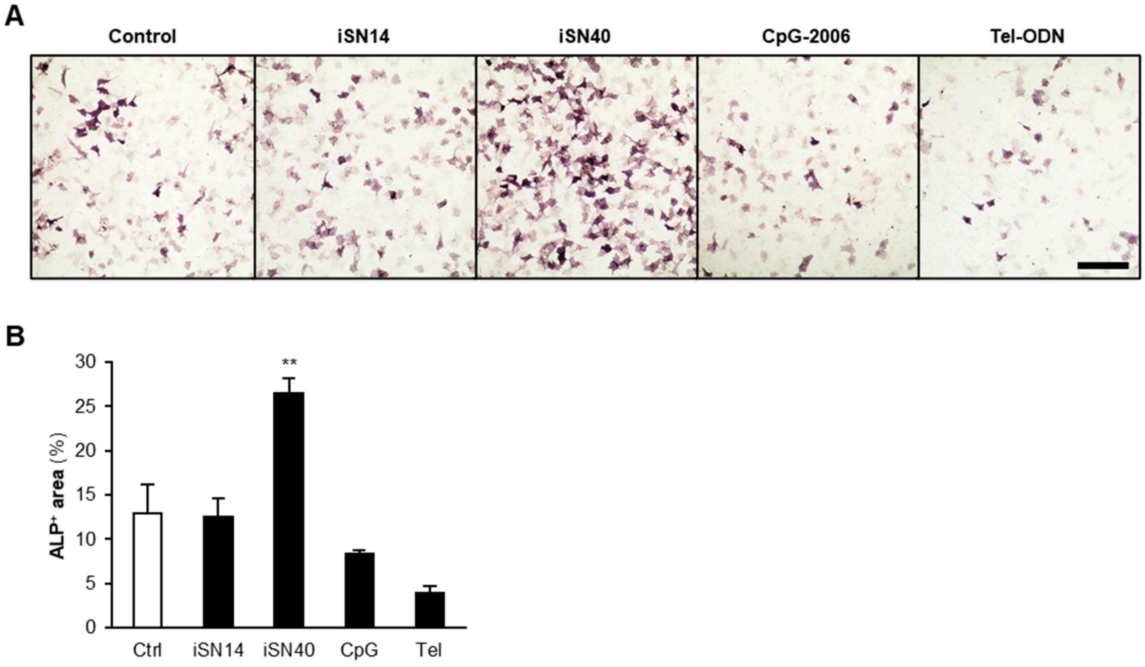

Figure S1. iSN40 enhances ALP activity in MC3T3-E1 cells. (**A**) Representative images of ALP staining of MC3T3-E1 cells treated with 10 μM PS-ODNs in GM for 48 h. Scale bar, 100 μm. (**B**) Quantification of ALP-positive area. ** $p < 0.01$ vs control (Scheffe's F-test). $n = 5$.

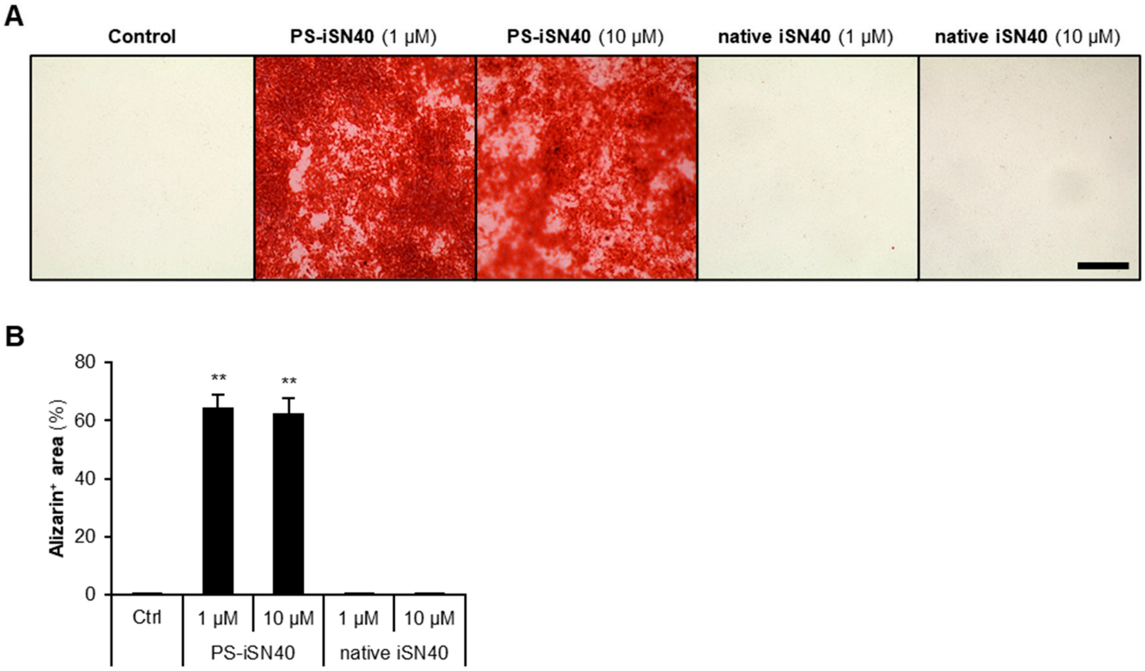

Figure S2. phosphorothioation is required for iSN40 to exert osteogenetic activity. (**A**) Representative images of alizarin staining of MC3T3-E1 cells treated with 1 or 10 μM of PS-iSN40 or native iSN40 in DM with 50 μg/ml AA for 10 days. Scale bar, 200 μm. (**B**) Quantification of alizarin-positive area. ** $p < 0.01$ vs control (Scheffe's F-test). $n = 4$.

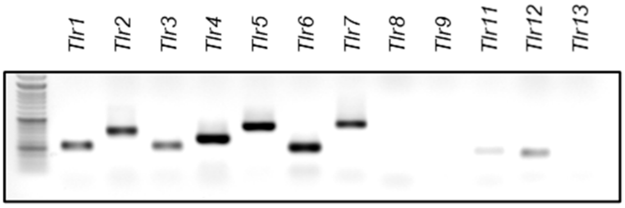

Figure S3. Expression of TLR genes in MC3T3-E1 cells. Total RNA of MC3T3-E1 cells maintained in GM was subjected to RT-PCR (40 cycles). Then the PCR products of TLR genes were subjected to agarose gel electrophoresis.

Table S1. ODN sequences.

Name	Sequence (5′-3′)	Reference
iSN04	AGATTAGGGTGAGGGTGA	[1]
iSN08	AGTTCAACATTAGGGTGA	[1]
iSN09	GTTCAACATTAGGGTGAA	[1]
iSN10	TTCAACATTAGGGTGAAA	[1]
iSN11	TCAACATTAGGGTGAAAA	[1]
iSN12	CAACATTAGGGTGAAAAT	[1]
iSN13	AACATTAGGGTGAAAATG	[1]
iSN14	ACATTAGGGTGAAAATGA	[1]
iSN15	CATTAGGGTGAAAATGAA	[1]
iSN16	TAAAGCATTAGGGTGATG	[1]
iSN17	AAAGCATTAGGGTGATGA	[1]
iSN18	AAGCATTAGGGTGATGAA	[1]
iSN19	AGCATTAGGGTGATGAAA	[1]
iSN20	GCATTAGGGTGATGAAAT	[1]

iSN21	CATTAGGGTGATGAAATC	[1]
iSN22	ATTAGGGTGATGAAATCC	[1]
iSN23	TTAGGGTGATGAAATCCA	[1]
iSN24	ATCAGGCTCAAGCTTGAG	[1]
iSN25	TCAGGCTCAAGCTTGAGT	[1]
iSN26	CAGGCTCAAGCTTGAGTT	[1]
iSN27	AGGCTCAAGCTTGAGTTC	[1]
iSN28	GGCTCAAGCTTGAGTTCT	[1]
iSN29	GCTCAAGCTTGAGTTGTG	[1]
iSN30	CTCAAGCTTGAGTTCTGA	[1]
iSN31	TCATTCCTAAGCTTGAGG	[1]
iSN32	CATTCCTAAGCTTGAGGC	[1]
iSN33	ATTCCTAAGCTTGAGGCC	[1]
iSN34	TTCCTAAGCTTGAGGCCT	[1]
iSN35	TCCTAAGCTTGAGGCCTA	[1]
iSN36	CCTAAGCTTGAGGCCTAT	[1]
iSN37	CTAAGCTTGAGGCCTATG	[1]
iSN38	TAAGCTTGAGGCCTATGG	[1]
iSN39	AAGCTTGAGGCCTATGGG	[1]
iSN40	GGAACGATCCTCAAGCTT	[1]
iSN40-GC	GGAAGCATCCTCAAGCTT	This study
iSN41	GAACGATCCTCAAGCTTA	[1]
iSN42	AACGATCCTCAAGCTTAG	[1]
iSN43	ACGATCCTCAAGCTTAGG	[1]
iSN44	CGATCCTCAAGCTTAGGT	[1]
iSN45	GATCCTCAAGCTTAGGTC	[1]
iSN46	TCCTCAAGCTTAGGTCCG	[1]
iSN47	CCTCAAGCTTAGGTCCGC	[1]
iSN48	AAATAGCTTTAGGGTTAG	[1]
iSN49	AATAGCTTTAGGGTTAGC	[1]
iSN50	ATAGCTTTAGGGTTAGCC	[1]
CpG-2006	TCGTCGTTTTGTCGTTTTGTCGTT	[2]
Tel-ODN	TTAGGGTTAGGGTTAGGGTTAGGG	[3]
MT01	ACCCCCTCTACCCCCTCTACCCCCTCT	[4]

Table S2. Primer sequences for RT-PCR.

Gene	Sequence (5′-3′)	Reference
Bglap2	GAACAGACTCCGGCGCTA<br>AGGGAGGATCAAGTCCCG	[5]
Col1a1	GCATGGCCAAGAAGACATCC<br>CCTCGGGTTTCCACGTCTC	[6]
Msx2	TGAGGAAACACAAGACCAA<br>GTCTATGGAAGGGGTAGGAT	[7]
Runx2	TTCTCCAACCCACGAATGCAC<br>CAGGTACGTGTGGTAGTGAGT	[8]
Sp7	TCCCTACCCAGCGCCCCACCTCT<br>CTGTGAATGGGCTTCTTCCTCAGC	[8]
Spp1	TGCCTGACCCATCTCAGAA<br>ATTCATCCGAGTCCACAGAA	[9]
Tlr1	TCTCTGAAGGCTTTGTCGATACA<br>GACAGAGCCTGTAAGCATATTCG	[10]
Tlr2	TCTAAAGTCGATCCGCGACAT<br>TACCCAGCTCGCTCACTACGT	[10]
Tlr3	TTGTCTTCTGCACGAACCTG<br>CGCAACGCAAGGATTTTATT	[10]
Tlr4	CAAGAACATAGATCTGAGCTTCAACCC<br>GCTGTCCAATAGGGAAGCTTTCTAGAG	[10]
Tlr5	ACTGAATTCCTTAAGCGACGTA<br>AGAAGATAAAGCCGTGCGAAA	[10]
Tlr6	AACAGGATACGGAGCCTTGA<br>CCAGGAAAGTCAGCTTCGTC	[10]
Tlr7	TTCCGATACGATGAATATGCACG<br>TGAGTTTGTCCAGAAGCCGTAAT	[10]
Tlr8	GGCACAACTCCCTTGTGATT<br>CATTTGGGTGCTGTTGTTTG	[10]
Tlr9	TGCAATTGGCTGTTCCTGAA<br>GGTGGTGGATACGGTTGGAG	[10]
Tlr11	CCAGGACTGCACCTTTTGG<br>GTGACACTGGTTGTACGCAAT	[10]
Tlr12	AGAGCTGGCTGGTATGTTCC<br>GTGTTCTTGTCAGGTCCAGAATC	[10]
Tlr13	GGAGCGCCTTGATCTAACTAACA<br>TCAGGTGGGTCAGAGAAACCA	[10]
Ywhaz	TTGATCCCCAATGCTTCGC<br>CAGCAACCTCGGCCAAGTAA	[11]

References

1. Nigar, S.; Yamamoto, Y.; Okajima, T.; Shigemori, S.; Sato, T.; Ogita, T.; Shimosato, T. Synergistic oligodeoxynucleotide strongly promotes CpG-induced interleukin-6 production. *BMC Immunol.* **2017**, *18*, 44.
2. Pohar, J.; Lainscek, D.; Fukui, R.; Yamamoto, C.; Miyake, K.; Jerala, R.; Bencina, M. Species-specific minimal sequence motif for oligodeoxyribonucleotides activating mouse TLR9. *J. Immunol.* **2015**, *195*, 4396–4405.
3. Sackesen, C.; van de Veen, W.; Akdis, M.; Soyer, O.; Zumkehr, J.; Ruckert, B.; Stanic, B.; Kalayci, O.; Alkan, S.S.; Gursel, I.; Akdis, C.A. Suppression of B-cell activation and IgE, IgA, IgG1 and IgG4 production by mammalian telomeric oligonucleotides. *Allergy* **2013**, *68*, 593–603.
4. Yang, G.; Wan, M.; Zhang, Y.; Sun, L.; Sun, R.; Hu, D.; Zhou, X.; Wang, L.; Wu, X.; Wang, L.; Yu, Y. Inhibition of a C-rich oligodeoxynucleotide on activation of immune cells in vitro and enhancement of antibody response in mice. *Immunology* **2010**, *131*, 501–512.
5. Deng, H.; Kuang, P.; Cui, H.; Luo, Q.; Liu, H.; Lu, Y.; Fang, J.; Zuo, Z.; Deng, J.; Li, Y.; Wang, X.; Zhao, L. Sodium fluoride induces apoptosis in mouse splenocytes by activating ROS-dependent NF-κB signaling. *Oncotarget* **2017**, *8*, 114428–114441.
6. Tian, Y.; Xu, Y.; Fu, Q.; Dong, Y. Osterix is required for Sonic hedgehog-induced osteoblastic MC3T3-E1 cell differentiation. *Cell Biochem. Biophys.* **2012**, *64*, 169–176.
7. Takayama, T.; Dai, J.; Tachi, K.; Shohara, R.; Kasai, H.; Imamura, K.; Yamano, S. The potential of stromal cell-derived factor-1 delivery using a collagen membrane for bone regeneration. *J. Biomater. Appl.* **2017**, *31*, 1049–1061.
8. Gao, J.; Feng, Z.; Wang, X.; Zeng, M.; Liu, J.; Han, S.; Xu, J.; Chen, L.; Cao, K.; Long, J.; Li, Z.; Shen, W.; Liu, J. SIRT3/SOD2 maintains osteoblast differentiation and bone formation by regulating mitochondrial stress. *Cell Death Differ.* **2018**, *25*, 229–240.
9. Lee, D.J.; Tseng, H.C.; Wong, S.W.; Wang, Z.; Deng, M.; Ko, C.C. Dopaminergic effects on in vitro osteogenesis. *Bone Res.* **2015**, *3*, 15020.
10. Shinji, S.; Umezawa, K.; Nihashi, Y.; Nakamura, S.; Shimosato, T.; Takaya, T. Identification of the myogenetic oligodeoxynucleotides (myoDNs) that promote differentiation of skeletal muscle myoblasts by targeting nucleolin. *Front. Cell Dev. Biol.* **2021**, *8*, 616706.
11. Veazey, K.J.; Colding, M.C. Selection of stable reference genes for quantitative rt-PCR comparisons of mouse embryonic and extra-embryonic stem cells. *PLoS One* **2011**, *6*, e27592.
